# Regulation and Function of the Atypical IκBs—Bcl‐3, IκB_NS_, and IκBζ—in Lymphocytes and Autoimmunity

**DOI:** 10.1002/eji.202451273

**Published:** 2025-05-13

**Authors:** Tanja Kübelbeck, Nina Olivera Wichmann, Timsse Raj, Cynthia Raj, Caspar Ohnmacht, Nadine Hövelmeyer, Daniela Kramer, Vigo Heissmeyer

**Affiliations:** ^1^ Department of Dermatology University Medical Center of the Johannes Gutenberg‐University of Mainz Mainz Germany; ^2^ Center of Allergy and Environment (ZAUM) Technical University and Helmholtz Zentrum München Munich Germany; ^3^ Institute for Immunology, Biomedical Center (BMC), Faculty of Medicine Ludwig‐Maximilians‐Universität in Munich Planegg‐Martinsried Germany; ^4^ Institute for Molecular Medicine Mainz University Medical Center of the Johannes Gutenberg‐University Mainz Mainz Germany; ^5^ Research Centre for Immunotherapy (FZI) University Medical Center of the Johannes Gutenberg‐University Mainz Mainz Germany; ^6^ Research Unit Molecular Immune Regulation Molecular Targets and Therapeutics Center Helmholtz Zentrum München Munich Germany

**Keywords:** autoimmunity, *Bcl3*, lymphocytes, NF‐κB, *Nfkbid*, *Nfkbiz*

## Abstract

Signaling pathways involving NF‐κB transcription factors have essential roles in inflammation, immunity, cell proliferation, differentiation, and survival. Classical IκB proteins, such as IκBα and IκBβ, bind to NF‐κB via ankyrin repeats to sequester NF‐κB in the cytoplasm and thus suppress NF‐κB activity. Unlike these constitutively expressed classical IκBs, the expression of the atypical IκBs Bcl‐3, IκB_NS_, and IκBζ is induced in immune cells after recognition of antigens, pathogen‐associated molecular patterns (PAMPs) or cytokines, upon which they localize to the nucleus and form complexes with transcription factors and regulators on the DNA. Atypical, nuclear IκBs have been proposed to modulate NF‐κB activity in a context‐dependent manner as they can either inhibit or increase gene expression of a subset of NF‐κB target genes. This complexity may be related to the molecular function of atypical IκBs, which bind to different transcription factor complexes and form a bridge to different cofactors or epigenetic modifiers. Recent research has identified novel target genes of atypical IκBs that include chemokines, cytokines, and master regulators of lymphocyte differentiation, underscoring prominent roles in adaptive immune and autoimmune responses. Here, we summarize our current understanding of atypical IκBs in lymphocytes with a focus on their emerging role in autoimmunity.

## Graphical abstract

This review article summarizes the currently available information on the functions of atypical IκB proteins including Bcl‐3, IκB_NS_, and IκBζ, which have been studied mostly in mouse models. We focus on the regulation of these modulators of transcription, describe their role in lymphocyte differentiation, and their involvement in the development of autoimmunity.

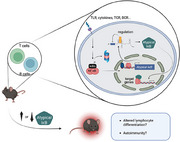

## 1 Introduction

**FIGURE 1:**
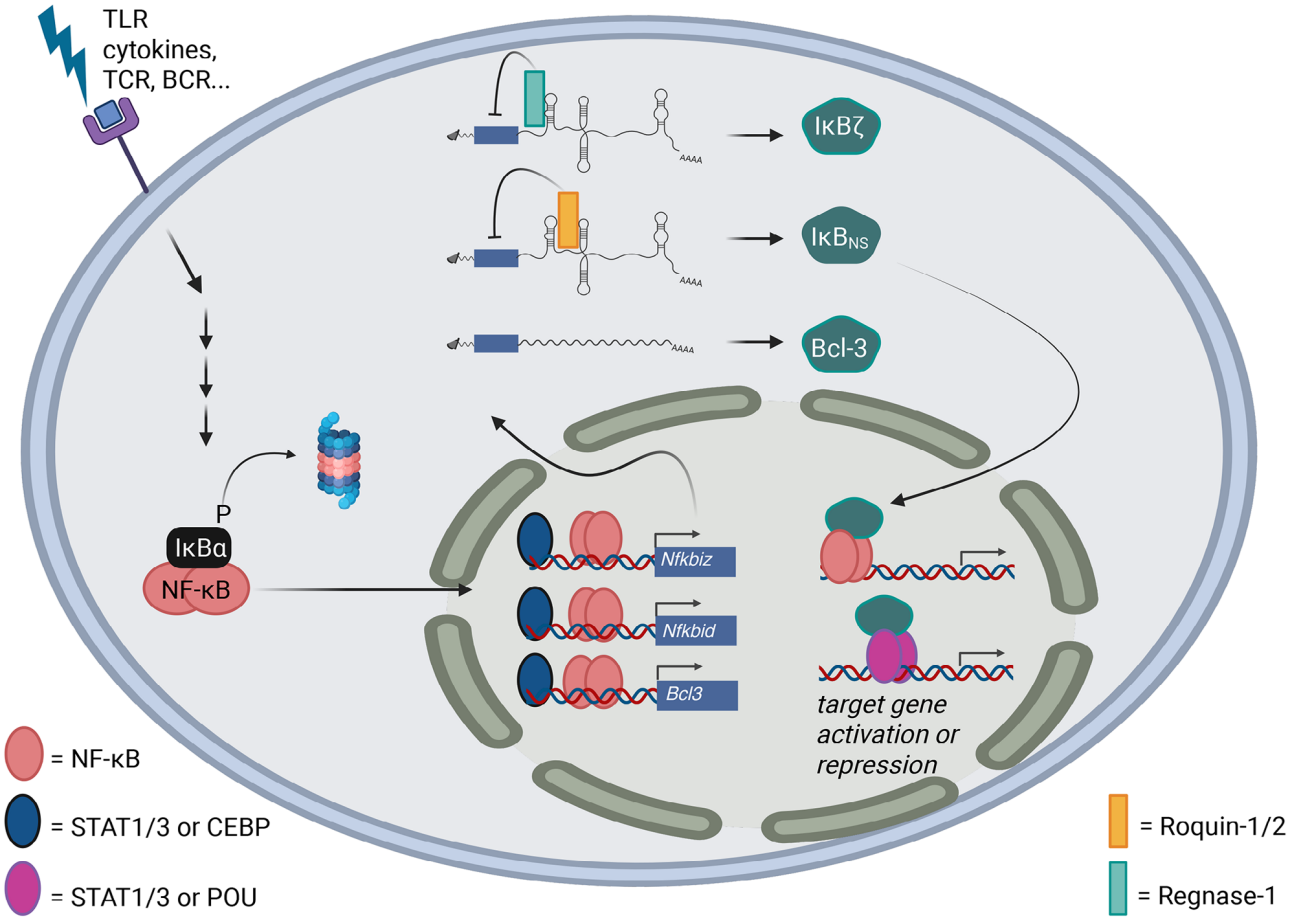
Overview of the regulation and function of the atypical IκB proteins, IκBζ (encoded by *Nfkbiz*), IκB_NS_ (encoded by *Nfkbid*), and Bcl‐3 (encoded by *Bcl3*). In resting cells, classical IκBs, such as IκBα sequester NF‐κB in the cytoplasm, rendering it inactive. Upon stimulation, NF‐κB is translocated into the nucleus. Subsequently, together with multiple other transcription factors such as STATs or CEBP, NF‐κB transcriptionally induces the expression of atypical IκBs. Moreover, Regnase‐1 and 3 as well as Roquin‐1 and 2 regulate the mRNA stability or expression of *Nfkbid* and *Nfkbiz*, thereby modifying the overall expression of IκBζ and IκB_NS_. Upon protein expression of the atypical IκBs, all three members associated with different transcription factor complexes, thereby recruiting other (epi)‐genetic co‐factors, changing chromatin accessibility, and ultimately gene expression of a subset of NF‐κB target genes. Please note that miRNA‐mediated regulation has been proposed for all three atypical IκBs, however, it is not displayed in this figure, since a physiologic relevance for lymphocyte function has not been demonstrated, yet.

NF‐κB constitutes a family of transcription factors that critically controls immune homeostasis. Thus, mutations and aberrations within the NF‐κB signaling pathway are associated with various autoinflammatory and autoimmune diseases [1, 2, 3]. As NF‐κB controls multiple functions, including survival, proliferation, differentiation, and activation of immune cells, its activity and effects on target gene expression have to be tightly controlled. IκB proteins comprise a key family of NF‐κB‐related co‐factors that not only regulate the overall activity of NF‐κB but also the expression of subgroups of NF‐κB target genes. This class of co‐factors can be roughly distinguished into classical IκBs such as IκBα and IκBβ, the precursor p105 and p100 that are processed to p50 and p52, respectively, and the atypical IκBs, such as Bcl‐3, IκBζ, and IκB_NS_. Whereas classical IκBs and the precursors p105 and p100 retain NF‐κB in the cytoplasm in the absence of NF‐κB signaling, the protein family of atypical IκBs, Bcl‐3 (encoded by *Bcl3*), IκBζ (encoded by *Nfkbiz*), and IκB_NS_ (encoded by *Nfkbid*), are inducibly expressed upon activation of NF‐κB (Figure 1). Subsequently, these atypical IκBs interact with NF‐κB, but also with other transcription factors on the chromatin to promote or suppress the transcriptional induction of a subset of NF‐κB target genes (Figure 1). Initial research on the atypical IκB proteins has compared them to classical IκBs and uncovered a preference for binding to p50 or p52 homodimers over p65/p50 heterodimers [4, 5, 6, 7, 8, 9, 10, 11]. While a cocrystal structure for the classical IκB, IκBα, in complex with NF‐κB p50/p65 has been solved [12], the interaction of atypical IκBs remains undefined at the structural level. Various investigations on the physiologic importance, interaction partners, and downstream function of atypical IκBs have been performed, but their exact role and mechanism of action are still poorly defined.

Given their flexible protein interactions, target gene regulation, and dynamic expression patterns, a direct comparison between studies is difficult. Thus, genetic models have been instrumental in understanding atypical IκB functions and underscore the prominent role of these molecules in lymphocytes and immune responses. Mouse knockout models of all three atypical IκBs (*Bcl3*, *Nfkbid*, and *Nfkbiz*) showed unperturbed development, but immune functions and cytokine production were altered already at homeostasis, during immune responses or in disease models [10, 13, 14, 15, 16, 17, 18]. The current challenge is to understand when and how the expression of Bcl‐3, IκB_NS,_ and IκBζ is induced, what functions these proteins have in different cell types, which genes they regulate, and how they impact the gene regulatory networks to control the differentiation of immune cells and affect the immune system or immune responses.

As a large part of our knowledge has been obtained in the mouse system, we will in this review, unless specified otherwise, refer to the mouse genes, the mRNAs encoded in the mouse, and functions of the proteins in the cells of the mouse immune system. A major focus of this review is placed on the role of atypical IκB in lymphocytes, trying to understand how these factors impact the development of immune‐related diseases and autoimmunity.

## 2 Molecular Features of Atypical IκB Proteins

**FIGURE 2:**
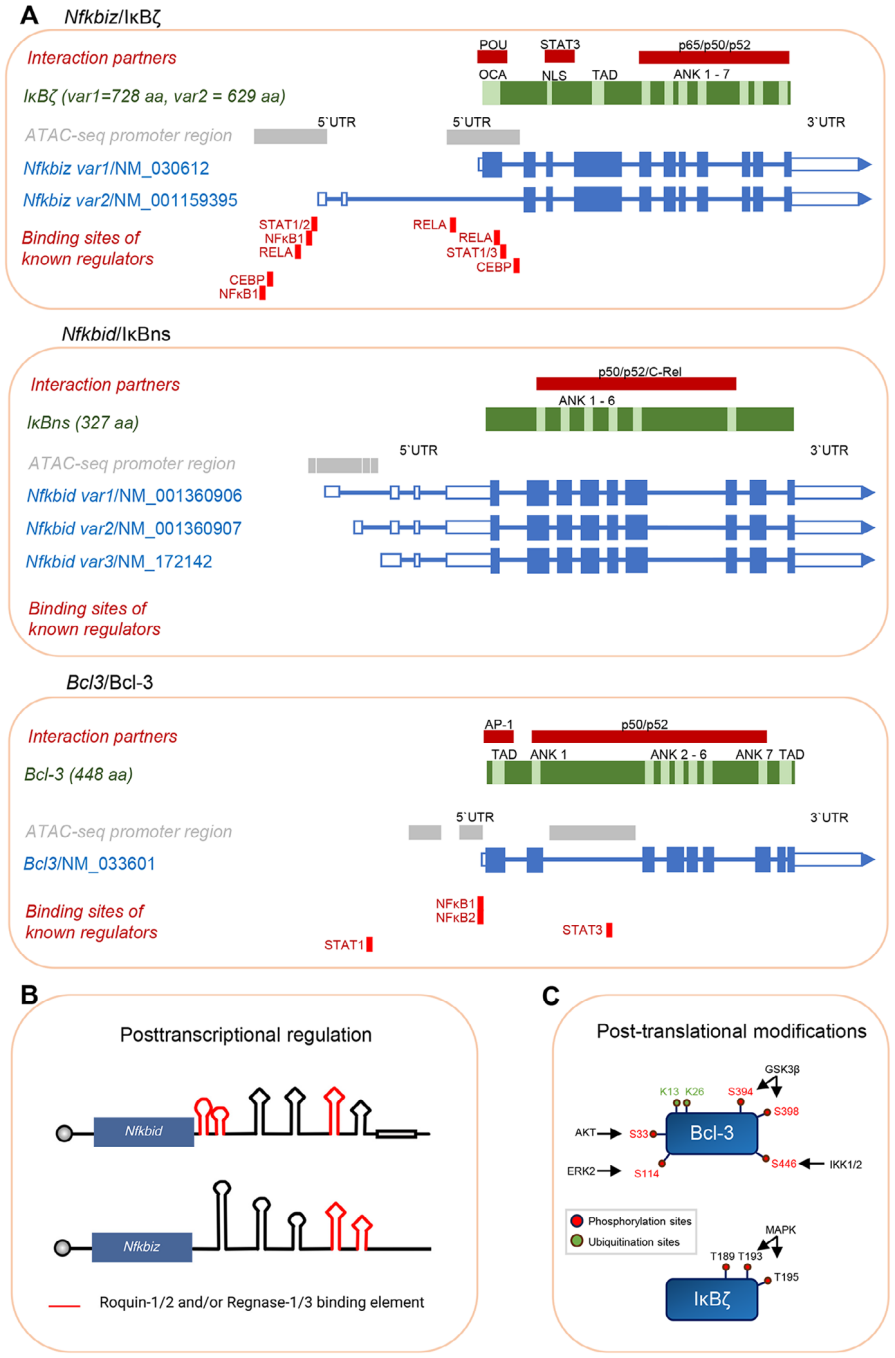
Gene structure, transcriptional and posttranscriptional regulation, and posttranslational modifications of Bcl‐3, IκB_NS,_ and IκBζ. (A) Gene structure. IκBζ is encoded by two different variants of *Nfkbiz*, resulting from alternative splicing, and thus generates two different proteins consisting of 728 and 629 amino acids. Of note, both variants contain unique promoter regions which can be occupied by canonical NF‐κB, STAT1‐3 or CEBP [126, 127, 128]. Besides 7 ankyrin repeats, it contains a nuclear localization signal (NLS), a potential TAD, and an OCA domain [19, 129]. Direct interaction and mapping of the interaction sites have already been identified for POU transcription factors [129], STAT3 [130], and NF‐κB p50, p52, and p65 [10, 131]. The smallest member of atypical IκBs is IκB_NS_, encoded by *Nfkbid*, which exists in three different isoforms through splicing events at the 5*'*‐UTR, leading to the generation of a 327 amino acid long protein. Our schematic omits a putative isoform of IκB_NS_ (470 aa) since this isoform has not been experimentally verified. It consists of 6 ankyrin repeats which mediate the interaction with NF‐κB p50, p52, and c‐Rel. Of note, no reporter promoter studies of *Nfkbid* have been published so far. Bcl‐3 (encoded by *Bcl3*) consists of 448 amino acids, and contains two transactivation domains (TAD) and 7 ankyrin (ANK) repeats [4, 132]. Moreover, several promoter studies revealed that Bcl‐3 expression is induced by binding of either canonical or noncanonical NF‐κB signaling [133], as well as STAT1 [134] and STAT3 [135]. Direct interactions of Bcl‐3 with AP1 [76] and p50/p52 have been reported [136, 137, 138]. (B) Posttranscriptional regulation. *Nfkbiz* and *Nfkbid*, but not *Bcl3*, are posttranscriptionally regulated by Regnase‐1 and 3 and Roquin‐1 and 2. Regnase and Roquin proteins can bind at the 3*'*‐UTR, thus inducing mRNA decay and inhibiting translation of IκBζ and IκB_NS_ at steady state [52, 59, 64]. (C) Posttranslational modifications. Apart from transcription and posttranscriptional regulation, posttranslational modifications of Bcl‐3 and IκBζ have been described, that modify their activity, binding to interaction partners, and overall function. Of note, no posttranslational modifications have been described for IκB_NS_ so far.

An overview of the genomic structure and regulation of all three atypical IκB proteins is shown in Figure 2. Of note, although multiple isoforms have already been described for the atypical IκBs, isoform‐specific functions have only rarely been investigated so far.

IκBζ, encoded by *Nfkbiz*, was originally identified as a protein called MAIL, short for molecule possessing ankyrin repeats induced by lipopolysaccharide. It was also termed INAP, which stands for interleukin (IL)‐1 inducible nuclear ankyrin‐repeat protein [19, 20]. The N‐terminus of IκBζ differs from the other IκB proteins [11], providing additional interaction sites with other transcription factors such as POU and STAT proteins. It also contains a transactivation domain (TAD), although the functionality of this domain remains under debate [21, 22]. Earlier publications suggested selective IκBζ complex formation with p50 and p52 homodimers, which themselves lack a TAD, consequently leading to an IκBζ‐dependent activation of NF‐κB target genes [22, 23, 24]. Other publications showed IκBζ binding to p65:p50 heterodimers and p50 homodimers, and involvement in the inhibition of DNA‐binding of these dimers [21, 25, 26]. IκBζ is expressed in nonhematopoietic cells such as keratinocytes, fibroblasts, and chondrocytes [19, 27‐30], as well as in innate and adaptive immune cells [10, 31‐36].

IκΒ_ΝS_, encoded by *Nfkbid*, is the smallest member of the atypical IκΒ protein family [6]. It contains six ankyrin repeats which interact with NF‐κB [37]. IκΒ_ΝS_ was originally identified in a study on negative selection (NS) in the thymus and has been suggested to interact with all members of the NF‐κB family in vitro [6]. Later studies determined p50 as the main interaction partner in macrophages [7] as well as p50:c‐Rel heterodimers in CD4^+^ T cells or Treg cells [9]. Promoter studies investigating regulators of IκΒ_ΝS_ expression are so far lacking, although NF‐κB is likely to be involved. IκΒ_ΝS_ has been described to be expressed in adaptive and innate immune cells and lung epithelial cells [7, 9, 13, 18, 38‐43].

Bcl‐3 (B cell leukemia 3 protein) is a proto‐oncogene that was originally identified by its translocation into the immunoglobulin alpha‐locus in some patients with chronic lymphocytic leukemia. It contains amino‐terminal and carboxy‐terminal TADs [44, 45]. The seven ankyrin repeats of Bcl‐3 interact with NF‐κB p50 or p52 homodimers and early studies suggested that Bcl‐3 represses the binding of these homodimers to DNA [4, 8, 46, 47]. Of note, many cell types express Bcl‐3 at a steady state, and its expression can be further induced by TLR activation in myeloid cells and other NF‐κB‐inducing agents. This process may partially depend on p50 expression [48].

## 3 Regulation

In most of the studied cell types, such as keratinocytes or T‐cells, *Nfkbiz* transcription, and IκBζ protein expression are induced in response to a variety of different NF‐κB‐ and STAT‐activating stimuli (Table 1), which is followed by rapid proteasomal degradation of the IκBζ protein [49, 50, 51]. The E3 ubiquitin ligase PDLIM2 may mediate IκBζ proteasomal degradation, at least in myeloid cells [51]. Moreover, multiple threonine phosphorylation sites have been identified in IκBζ, which can switch IκBζ from a gene activator to a gene repressor by promoting the recruitment of HDAC1 to target gene promoters [21] (Figure 2C). The *Nfkbiz* mRNA is also posttranscriptionally regulated by the endoribonuclease activities of Regnase‐1 and Regnase‐3 which bind to a defined *cis*‐element composed of several stem‐loop structures in the 3*'*‐UTR of *Nfkbiz* and induce mRNA decay but also translational inhibition [52, 53, 54, 55, 56] (Figure 2B). The inhibitory effect of Regnase‐1 can also be counteracted by Arid5a, which, during IL‐17 stimulation, seems to interfere with Regnase‐1 activity, stabilizes *Nfkbiz* mRNA, and promotes IκBζ protein expression [57]. Regnase‐1 is a target of MALT1 proteolytic cleavage [58], and *Nfkbiz*/IκBζ expression is strongly triggered by MALT1 activation in T cells [59]. Moreover, treatment of macrophages and keratinocytes with itaconate suppressed LPS‐induced IκBζ expression, possibly through ATF3‐dependent repression [60]. MicroRNAs also regulate *NFKBIZ* mRNA stability. In detail, miR‐376b and miR‐124a can inhibit *NFKBIZ* expression, while miR‐376b and *Nfkbiz have* an impact on liver regeneration, tubular damage, and intrarenal inflammation in acute kidney injury [61, 62, 63].

**TABLE 1:** Expression stimuli, target genes, and interaction partners of atypical IκBs.

	IκBζ	IκΒ_ΝS_	Bcl‐3
Stimulus and receptor leading to expression	B‐cell: BCR [35]	B‐cell: Tlr1/2 (Pam3CSK4) [108]Tlr4 (LPS) [108] Tlr6/2 (FSC‐1) [108] Tlr7 (Imiquimod) [108] Tlr9 (ODN1826) [108] BCR [100, 103]	
	T‐cell: Il6r (IL‐6) [36] Tgfbr (TGF‐β) [36]	T‐cell: TCR (IL‐2, CD3, and CD28) [9, 59, 70]	
Target genes	B‐cell: *Il10* [35], *Ctla4* [35], *Cd86* [35], *Tnfa* [35, 99]	B‐cell: *Pax5* [106], *Blimp1* [106, 107]	
	T‐cell: *Il17a* [36]*, Il17f* [36]*, Il21* [36]*, Il22* [36]*, Il23r* [36] Treg: *Foxp3* [82]	T‐cell: *Bcl6* [85], *Il2* [18, 83], *Il10* [83], *Ccl3* [83], *Csf2* [18, 83], *Il17f* [84], *Ccr6* [84] Treg: *Foxp3* [9]	T‐cell: *Gata3* [93] *Rorc* [94] *Bim* [90] Treg: *Ctla4* [92], *Foxp3* [92], *Il2r* [92], *Il10* [92]
Interaction partner	p50, p65 [26]—in macrophages Akirin2 [139]—in macrophages POU transcription factors [129]—HEK293T C/EBPβ, STAT3, STAT1 [127]—in HaCaT AP1, KLF4 [126]—in HaCaT PDLIM2 [51]—in macrophages RORγt and RORα [36]—in CD4^+^‐T‐cells (some experiments Th17 condition) Foxp3 [82]—in EL‐4/LAF	p50 [7]—in macrophages p65, Rel, Relb [6]—in thymic extracts from N15 TCRtg mice cRel, p50 [9]—in CD4^+^CD25^‐^ Tcon	p50 [92]—in Tregs p52 [140]—in macrophages HDAC1 [48]—in macrophages

IκΒ_ΝS_ protein expression and *Nfkbid* transcription are induced by several stimuli including antigen receptors, TLRs, or cytokine receptors (Table 1), but the *Nfkbid* mRNA is also placed under profound posttranscriptional control. The 3*'*‐UTR of the *Nfkbid* mRNA harbors several stem–loop structures that form a *cis*‐element [64], very similar to the one defined in *Nfkbiz* [52] (Figure 2B). Both *cis*‐elements contain one or two constitutive decay elements (CDEs), which are known to be recognized by the Roquin family of RNA‐binding proteins, namely Roquin‐1 and its redundantly functioning paralog Roquin‐2 [52, 64‐68]. *Nfkbid*/IκΒ_ΝS_ mRNA and protein expression are regulated by Roquin‐1 via induced mRNA decay that involves deadenylation and decapping as well as inhibition of translation [64]. Very similar to IκBζ, IκΒ_ΝS_ is strongly derepressed upon activation of the MALT1 protease, which is explained by Roquin‐1 and Roquin‐2 being MALT1 substrates [59, 69]. In fact, IκΒ_ΝS_ induction upon TCR signaling closely follows the proteolytic cleavage of Roquin proteins and is prevented by MALT1 inhibition or mutations of the MALT1‐specific cleavage sites in Roquin‐1 [59, 70]. *NFKBID* is also regulated by microRNA. It has been shown that miR‐492 binds *NFKBID* and leads to the downregulation of *NFKBID* mRNA levels in the context of Zika virus replication [71].

Bcl‐3 protein levels are regulated via posttranslational modifications, especially phosphorylation and ubiquitination (Figure 2C). Under resting conditions, Bcl‐3 degradation and oncogenicity are regulated by protein kinase GSK3β‐mediated phosphorylation [72, 73]. However, the proteasomal degradation in the cytoplasm was shown to be independent of GSK3 [74]. The ability of Bcl‐3 to affect gene transcription depends on its phosphorylation by AKT, ERK2, and IKK1/2, which enable the regulation of NF‐κB p52 and p50 homodimer transcriptional activity [73]. Translocation from the cytoplasm to the nucleus requires K‐63‐linked polyubiquitination [73]. The deubiquitinating enzyme CYLD has been shown to control Bcl‐3 localization in keratinocytes by removing polyubiquitin chains upon UV irradiation. This prevents nuclear accumulation and consequently Bcl‐3‐mediated regulation of gene transcription [75]. Bcl‐3 can suppress transcription by recruiting transcriptional co‐repressors. This was specifically demonstrated by the recruitment of HDAC1 and the subsequent suppression of TNF production in macrophages upon LPS stimulation [48]. The recruitment of the co‐repressor CtBP by Bcl‐3 was associated with increased Bcl‐3 stability and enhancement of its suppressive capacity [74]. Bcl‐3 can also activate gene transcription by forming a ternary complex with p50 homodimers, inducing transcription through its TADs [4]. The interaction of Bcl‐3 with proteins such as histone acetyltransferases (e.g., p300 and Tip60) suggests, among others, a putative role of Bcl‐3 in chromatin remodeling [76, 77]. Lastly, Bcl‐3 expression may also be repressed by the microRNAs miR‐125b and miR‐19a [78, 79] but functional investigations for the relevance of these observations in noncancerous cells are still lacking.

## 4 Function in T Cells

IκBζ is highly expressed in Th17 cells compared with other T helper cell subsets. Combined IL‐6 and TGF‐β stimulation triggers its induction in Th17 cells, which depends on STAT3 [36]. While CD4^+^ T cells isolated from global *Nfkbiz* knockout mice exhibit normal Th17 differentiation, they completely lose IL‐17A expression in a RORγt and RORα‐dependent manner (Table 1). Consequently, no experimental autoimmune encephalomyelitis (EAE) was induced when CD4^+^ T cells from global *Nfkbiz* knockout mice were transferred into *Rag2* knockout mice [36]. Furthermore, MaruYama et al. [80] explored the role of IκBζ in T cells using Lck‐Cre *Nfkbiz* knockout mice. These mice developed lymphadenopathy, splenomegaly, and leukocyte infiltration in various tissues and organs between 6‐18 months of age. In younger mice, deletion of *Nfkbiz* resulted in increased numbers of Treg cells and effector/memory CD4^+^ T cells as well as increased serum levels of IFN‐γ and IL‐2. These effects may be partly due to the use of the Lck‐Cre, as a report implied adverse and off‐target effects in the Lck‐Cre line, and wild‐type mice without Lck‐Cre were used as controls [81]. Opposingly, a Treg‐specific knockout of *Nfkbiz* did not show significant differences in the numbers of effector T cells, thymic‐derived Treg cells, or expression levels of key cytokines [80]. In contrast, Treg cells from Lck‐Cre *Nfkbiz* knockout mice exhibited reduced immunoregulatory function in a T‐cell transfer colitis model. Further studies suggested that IκBζ can bind to the Foxp3 promoter in the presence of TGF‐β in Treg cells and can inhibit Foxp3 expression by interfering with p65 transactivation [82], thereby possibly interfering with Treg differentiation or function (Table 1).

IκΒ_NS_ is expressed in both effector and regulatory T cells [9, 18, 83]. Although global *Nfkbid* knockout mice did not show changes in immune cell populations within the thymus or peripheral lymphoid organs, conditional inactivation reveals a proliferation defect of IκΒ_ΝS_‐deficient CD4^+^ and CD8^+^ T cells in vitro, which can be rescued by exogenous IL‐2 and IL‐7 supplementation [13, 18]. IκΒ_ΝS_‐deficient T cells expressed lower levels of IL‐2 and IFN‐γ [18]. They also showed a specific impairment in the induction of RORγt in response to TGFβ and IL‐6 and were less capable of differentiating into the Th17 subset, exhibiting reduced expression of IL‐17A and Th17‐related genes as compared with wild‐type counterparts [84] (Table 1). *Citrobacter rodentium* infections revealed that the absence of IκΒ_ΝS_ significantly reduced the infiltration of IL‐17A^+^ T‐cells into the gut lamina propria [83]. IκΒ_ΝS_ does not directly regulate *Il17a* transcription, but instead interacts with the *Il10* gene locus, as shown by chromatin immunoprecipitation (ChIP) [83, 84] (Table 1). In *Listeria monocytogenes* (*L.m*.) infections IκΒ_ΝS_ was required for the induction of *L.m*.–Ova‐specific Th1 cells and effector cytokine production. Although IκΒ_ΝS_ expression was necessary during the early stages of Th1 priming, it did not affect T‐bet expression [39]. Additional roles for IκΒ_ΝS_ in promoting Tfh cell differentiation were described and direct regulation of the Tfh signature genes, *Bcl6* and *Il21*, was confirmed in ChIP experiments [85]. IκΒ_ΝS_ also plays a critical role in thymic Treg development. In ChIP experiments IκΒ_ΝS_ bound to the conserved noncoding region 3 (CNS3) in the *Foxp3* promoter via p50 and c‐Rel, and IκΒ_ΝS_ was required for the induction of Foxp3 but dispensable for CD25 expression [86] (Table 1). *Nfkbid*‐deficient Treg cells accumulated in the GITR^+^ CD25^+^ Foxp3^–^ precursor stage, failing to progress into mature thymic Treg cells, which caused a 50% reduction in mature peripheral Treg cells [9]. Functionally, *Nfkbid*‐deficient Treg cells were unable to protect from T‐cell transfer‐induced colitis [9]. In mature Treg cells, *Nfkbid* is suppressed by Foxp3 and is not required for the maintenance or suppressive function of Treg cells [9, 87].

Bcl‐3 was shown early on to be highly expressed in tolerogenic T cells and to directly control the formation of NF‐κB dimers and IL‐2 production [88]. Bcl‐3 was proposed to slow down T cell activation early after stimulation by a T cell‐intrinsic mechanism [89]. Like its function in other cell types, Bcl‐3 controls survival and apoptosis following activation of T cells: overexpression of Bcl‐3 increases survival, while Bcl‐3 deficiency accelerates cell death. The anti‐apoptotic activity of Bcl‐3 is partially based on the inhibition of the proapoptotic molecule Bim, since Bim was overactivated in Bcl‐3‐deficient T cells, and forced Bcl‐3 expression kept T cells alive but failed to promote the survival of Bim‐deficient T cells [90] (Table 1). In line with these results, mixed bone marrow chimeras showed that Bcl‐3‐deficient thymocytes were outcompeted by wild‐type cells; however, this effect was completely reversed in the intestinal lamina propria, presumably due to high amounts of Th17 and Treg cells at this site, and the strong effects of Bcl‐3 in restraining these cells [91]. Importantly, CD4^+^ T cells overexpressing Bcl‐3 fail to induce colitis in a T cell transfer‐induced colitis experiment, presumably due to impaired proliferation of these cells in vivo, which is a prerequisite for inducing colitis in this model [92]. Bcl‐3 has unique roles in different T helper cell subsets. For example, in vitro differentiation of Th2, but not Th1 cells, is impaired in Bcl‐3‐deficient T cells [93], as Bcl‐3 together with p50 transactivates the *GATA3* promoter [93] (Table 1). In Th1 cells, Bcl‐3 expression suppresses trans‐differentiation toward less pathogenic Th17‐like cells [94]. Mechanistically, Bcl‐3 prevents the binding of c‐Rel and p50 at the *RORC* locus and Bcl‐3‐deficient Th1 cells already show higher *Rorc* expression [94] (Table 1). Similarly, Bcl‐3‐deficient animals harbor elevated frequencies of (nonpathogenic) Th17 cells in the lamina propria of the small intestine, while overexpression of Bcl‐3 in T cells results in impaired Th17 differentiation in vitro and reduced frequencies of Th17 cells in the lamina propria of the small intestine [91, 95]. Pathogenicity of Th17 cells, indicated by co‐expression of IFN‐γ and GM‐CSF, may be regulated by Bcl‐3 at the metabolic level because enhanced glycolysis and lower respiration are observed in Bcl‐3‐deficient Th17 cells [96]. Bcl‐3 directly interacts with Raptor, one of the mTORC1 components, to control cell metabolism of Th17 cells [96]. Noteworthy, Bcl‐3‐deficient animals harbor elevated numbers of Treg cells in various compartments whereas T‐cell‐specific overexpression of Bcl‐3 results in impaired Treg cell differentiation and function [91, 92]. This is not only true for thymic‐derived Treg cells, but also for microbiome‐induced Treg cells co‐expressing RORγt [91, 97]. Additionally, there is also some evidence that Bcl‐3 affects the differentiation of CD8^+^ T cells, thereby limiting terminal effector cell differentiation and promoting memory cell formation [98].

## 5 Function in B cells

IκBζ expression is induced in B cells by activation of the BCR and/or by co‐stimulation with TLR9/TLR7 [35, 99]. IκBζ‐deficient mice showed impaired proliferation of B cells after TLR9 stimulation compared with wild‐type mice, but not after BCR stimulation. There were no differences in the number of mature B cells, follicular B cells, and the expression of surface markers such as IgM, FcγRIIB, and TLR9 in *Nfkbiz*‐deficient mice. In contrast, a slightly reduced number of transitional B cells and a slightly increased number of marginal zone B cells were present in these mice. Likewise, no difference in NF‐κB activation of IκBζ‐deficient B cells could be detected after TLR9 stimulation [35].

However, individual genes show differences in expression in IκBζ‐deficient B cells, including *Il10*, *Ctla4*, *Tnf*, and *Cd86* expression, although some of these effects were stimulus‐dependent (Table 1) [35].

IκB_NS_ expression is rapidly induced in B cells at the mRNA and protein level in response to LPS, anti‐CD40, and anti‐IgM stimulation [38, 100]. IκΒ_ΝS_‐deficient mice, or mice that harbor a premature stop codon in the *Nfkbid* gene, also known as *bumble* mice, completely lack B1 cells, show a delayed IgG response, and fail to differentiate toward plasma cells [38, 101‐103]. Additionally, *bumble* mice have severely reduced marginal zone (MZ) B cells and reduced IgM levels in the circulation. The MZ B cell compartment was restored to normal levels in aged bumble mice; however, these cells were not functional [104]. Interestingly, mice carrying a heterozygous *bumble* mutation also showed reduced IgM production despite having normal B cell development, which suggests a requirement for full IκΒ_ΝS_ expression from two *Nfkbid* alleles [105]. Furthermore, IκΒ_ΝS_ was shown to be required for the generation of plasma blasts and plasma cells in response to LPS stimulation. In *bumble* B cells, expression of *Pax5* and *Blimp1* which regulate plasma cell differentiation is increased (Table 1). This is accompanied by an excessive metabolic activity observed in *bumble* B cells that leads to impaired T‐cell‐independent antibody responses [106, 107]. Similar to T cells, IκΒ_ΝS_ was required for IL‐10 production by B cells following TLR stimulation, at least during the initial phases of induction, with IL‐10 expression normalizing over time [108].

Bcl‐3‐deficient mice show a requirement for Bcl‐3 in the germinal center reaction and immunization‐induced antibody responses [15, 109]. The diminished humoral immune responses may explain why Bcl‐3‐deficient mice show impaired clearance of *Listeria monocytogenes*, *Streptococcus pneumoniae*, and *Toxoplasma* infections [14, 15, 109]. In contrast, the Eμ‐*Bcl3* transgenic mice, in which Bcl‐3 is overexpressed in B and T cells, display splenomegaly and an accumulation of mature B‐cells in secondary lymphoid organs [110]. Transgenic mice that overexpress or lack Bcl‐3 specifically in B cells show that Bcl‐3 is a pivotal regulator of B cell fate determination. Loss of Bcl‐3 leads to an increase in marginal zone B cells and a reduction in follicular B cells, whereas overexpression of Bcl‐3 causes the opposite phenotype [111, 112]. Furthermore, Bcl‐3 promotes the survival and proliferation of B cell receptor‐stimulated B cells while impairing responses to LPS [112]. Bcl‐3 modulates the growth capacity of B cells, where its overexpression is linked to reduced proliferation upon activation, likely due to decreased cell death rather than increased growth [111, 112]. Taken together, Bcl‐3 possesses antiapoptotic properties that are critical for safeguarding B cells from programmed cell death.

## 6 Atypical IκBs in Autoimmunity and Autoinflammation

Given their importance in B and T cells, it is plausible that atypical IκBs play pivotal roles in spontaneous autoimmunity or experimental models of brain or gut autoimmunity and autoinflammation.

### 6.1 Spontaneous Disease

Global *Nfkbiz‐*deficient mice show 90% lethality during embryogenesis. 4–8‐week‐old mice develop lesions resembling atopic dermatitis that affect the face and neck and are characterized by strong infiltration of leukocytes [16, 113]. This phenotype has been related to Sjögren's syndrome and is associated with enhanced apoptosis due to IκBζ deficiency in epithelial cells of the lacrimal gland as well as the formation of Sjögren's syndrome‐associated autoantibodies in the serum of *Nfkbiz* knockout mice [28]. Interestingly, the salivary glands are also affected, since female *Nfkbiz*‐deficient mice show a reduced salivary flow rate, which was associated with dysbiotic oral microbiota and focal lymphocytic sialadenitis [114].

Global *Nfkbid*‐deficient mice or mice deficient in both IκΒ_ΝS_ and c‐Rel do not show any signs of autoimmunity or severe abnormalities in the development of the immune system, despite having a reduced Treg cell compartment [18]. The lack of autoimmunity has been attributed to the impaired activation of conventional T‐cells, which may balance the loss of Treg cells [115]. Interestingly, NOD mice express a hypermorphic *Nfkbid* allele which leads to a reduction in negative selection of diabetogenic CD8^+^ cells and may therefore contribute to autoimmune diabetes [116].

Global *Bcl3* knockout mice also do not show signs of autoimmunity, whereas the combined absence of *Bcl3* and *Nfkb2* results in the loss of central tolerance and autoimmunity [117]. This phenotype manifests despite the presence of elevated numbers of Treg cells in the context of Bcl‐3 deficiency. Although *Bcl3*‐deficient mice do not suffer from spontaneous autoimmunity, they are more susceptible to streptozotocin‐induced type 1 diabetes [118]. Also, in the context of systemic lupus erythematosus (SLE)–like disease, Bcl‐3 was shown to play a protective role, since *Bcl3*‐deficient mice carrying the *lpr* mutation developed a more severe SLE–like inflammatory phenotype than control *lpr* mice [119]. In line with this observation, forced overexpression of Bcl‐3 in T cells impaired Treg cell development and function, resulting in a spontaneous colitis phenotype [92]. Even though not related to autoimmunity, *Bcl3* deficiency also resulted in resistance to skeletal muscle atrophy, a phenomenon that is phenotypically mimicked in p105/p50 (*Nfkb1*) knockout mice [120].

### 6.2 Experimental Autoimmune Encephalomyelitis

Resistance to EAE has been reported for *Nfkbid* and *Nfkbiz* knockout mice; in both cases, the phenotype is T‐cell‐intrinsic and can be explained by reduced Th17 differentiation [36, 84]. Surprisingly, both Bcl‐3 deficiency in T cells and conditional overexpression of Bcl‐3 in CD4^+^ T cells protect mice from the development of EAE [94, 95]. This protection is associated with a strong reduction of immune cells infiltrating the central nervous system of both models. Resistance to EAE can be explained by the trans‐differentiation of Th1 cells to nonpathogenic Th17 cells in Bcl‐3‐deficient mice, whereas T cells overexpressing Bcl‐3 fail to differentiate into pathogenic Th17 cells [94, 95].

### 6.3 Gut‐Related inflammation

Global *Nfkbiz* knockout mice exhibit greater weight loss after dextran sulfate sodium (DSS) treatment and develop more severe colitis as determined by histopathological analysis [121]. However, mice with epithelial‐specific deletion of *Nfkbiz* (Vil1‐Cre) show similar levels of DSS‐induced inflammation as control mice, which suggests that the observed phenotype is driven by immune rather than epithelial cells [122]. Consistently, it has been shown that in patients with ulcerative colitis, the inflamed gut is remodeled by pervasive clones. Many of these clones are positively selected by the acquisition of mutations, often affecting the *NFKBIZ* gene. Consequently, these mutations are involved in the downregulation of IL‐17 signaling, and proinflammatory signaling [122, 123, 124]. In contrast, global *Bcl3*‐knockout mice are less susceptible than wild‐type mice to DSS‐induced colitis. The absence of Bcl‐3 was associated in one study with enhanced epithelial cell turnover and regeneration, despite similar levels of inflammation compared with wild‐type counterparts [125], whereas in another study, protection from DSS‐induced colitis was associated with elevated frequencies of RORγt‐expressing Treg cells [97]. Mice with a T‐cell‐specific overexpression of Bcl‐3 develop more severe colitis that can be attributed to defective Treg cell development and function [92].

In a transfer‐induced colitis model, *Rag1* ^–/–^ mice receiving *Nfkbid* knockout T cells developed a more severe form of colitis, characterized by an increase in IFN‐γ^+^ T cells and a complete loss of IL‐17A‐producing cells [83]. In contrast, *Rag1* ^–/–^ mice that received Bcl‐3‐deficient T cells were protected from transfer‐induced colitis. Upon transfer of naïve T cells into *Rag1* ^–/–^ recipients, Bcl‐3 deficiency resulted in a preferential differentiation to nonpathogenic Th17 cells and a reduced differentiation toward pathogenic Th1 cells and/or further trans‐differentiation of Th1 cells into nonpathogenic Th17 cells [94]. Additionally, Bcl‐3‐deficient T cells may preferentially differentiate toward RORγt^+^ Treg cells, with increased frequencies of both thymic and microbiome‐induced RORγt^+^ Treg cells potentially contributing to protection against colitis [91, 97].

In summary, all these observations suggest an important contribution of all three atypical IκBs, Bcl‐3, IκB_NS_, and IκBζ, in the differentiation and functionality of T and B cells. The impact of these molecules is controlled and finetuned at the transcriptional, posttranscriptional, and posttranslational levels all together enabling the time‐ and context‐dependent regulation of adaptive immune cells. Thus, we propose that atypical IκBs serve to enhance flexibility for the use of NF‐κB‐dependent gene regulation and harmonize this functionality with other pathways such as control of chromatin accessibility or cell metabolism with—if disturbed—important consequences for autoimmune disorders and intestinal inflammation.

## 7 Conclusion and Outlook

The investigation of atypical IκBs began with gene identification, followed by characterization of the gene products, examination of their regulation, and generation of mouse models with global or conditional knockouts or overexpression. The various approaches have generated numerous indications that implicate these factors in the control of T and B cell activation, differentiation, and survival and the development of autoimmune diseases. However, despite the use of similar approaches and models, in many cases, differing experimental setups preclude direct comparisons. Consequently, fundamental questions remain unanswered. For example, is the involvement of IκB_NS,_ and IκBζ in the same cell types and phenotypes and in the same molecular processes explained by redundant or cooperative functions? Which target genes are bound by individual atypical IκBs in a specific cell type? Which NF‐κB homo‐ or heterodimers are bound by these atypical IκBs in cells? Through which of the proposed mechanisms do they regulate the expression of specific target genes?

In future research, it will be crucial to reassess specific functions in parallel using conditional inactivation of floxed alleles with the same Cre lines and to generate double knockout models that can answer questions about redundancy. The studies of atypical IκBs in T cells have focused more on T helper cells, therefore additional studies will now be required to describe their role in cytotoxic T cells during infections to provide information for a more balanced view. We propose that biochemical and bioinformatic approaches to determine genome‐wide interactions will be essential for advancing our understanding. Specifically, developing comprehensive and comparative genome‐wide binding maps for Bcl‐3, IκB_NS_, and IκBζ in the same cell type, using Cut&Run or Cut&Tag technologies would be an invaluable resource to uncover the function of atypical IκBs, especially in lymphocytes and in the setting of autoimmunity and autoinflammatory diseases.

## Conflicts of Interest

The authors declare no conflicts of interest.

### Peer Review

The peer review history for this article is available at https://publons.com/publon/10.1002/eji.202451273.

## Data Availability

Data sharing is not applicable to this article as no datasets were generated or analyzed during the current study.
